# Supplementary material for: Single-cell transcriptome analysis reveals cellular heterogeneity in mouse intra- and extra articular ligaments
Source: Commun Biol. 2022 Nov 12;5:1233. doi: 10.1038/s42003-022-04196-w (PMC9653455; doi:10.1038/s42003-022-04196-w)
Supplement: Supplementary file 2 — Supplementary information [file 42003_2022_4196_MOESM2_ESM.pdf]

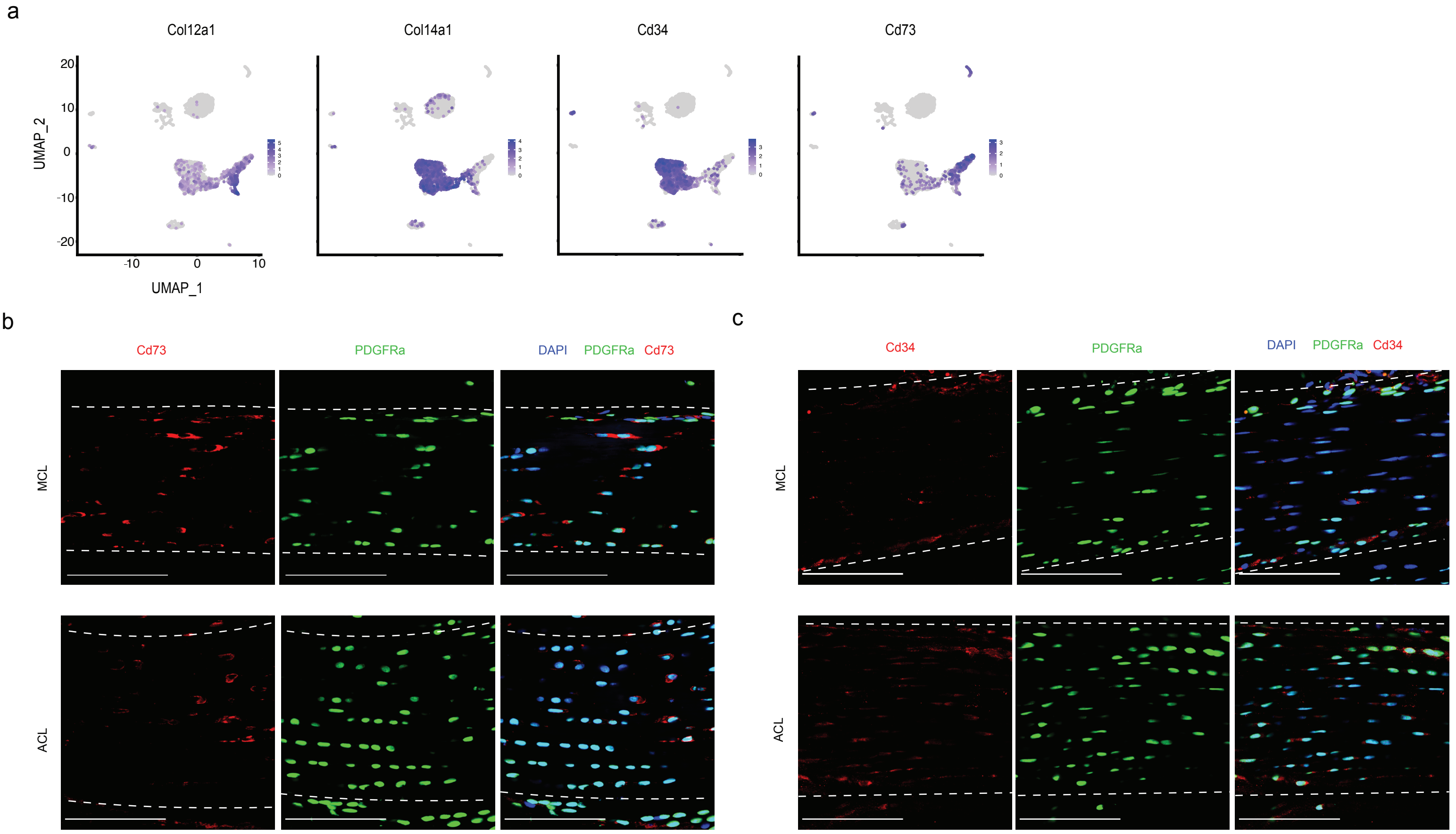

Supplementary Fig. 1. Identification of Col12a1- and Col14a1-positive fibroblasts

a UMAP plots showing the expression of *Col12a1*, *Col14a1*, *Cd34* and *Cd73,* in the ACL and MCL. b,c Immunostaining of Cd73 (b) and Cd34 (c) using PDGFRaH2BGFP mice.

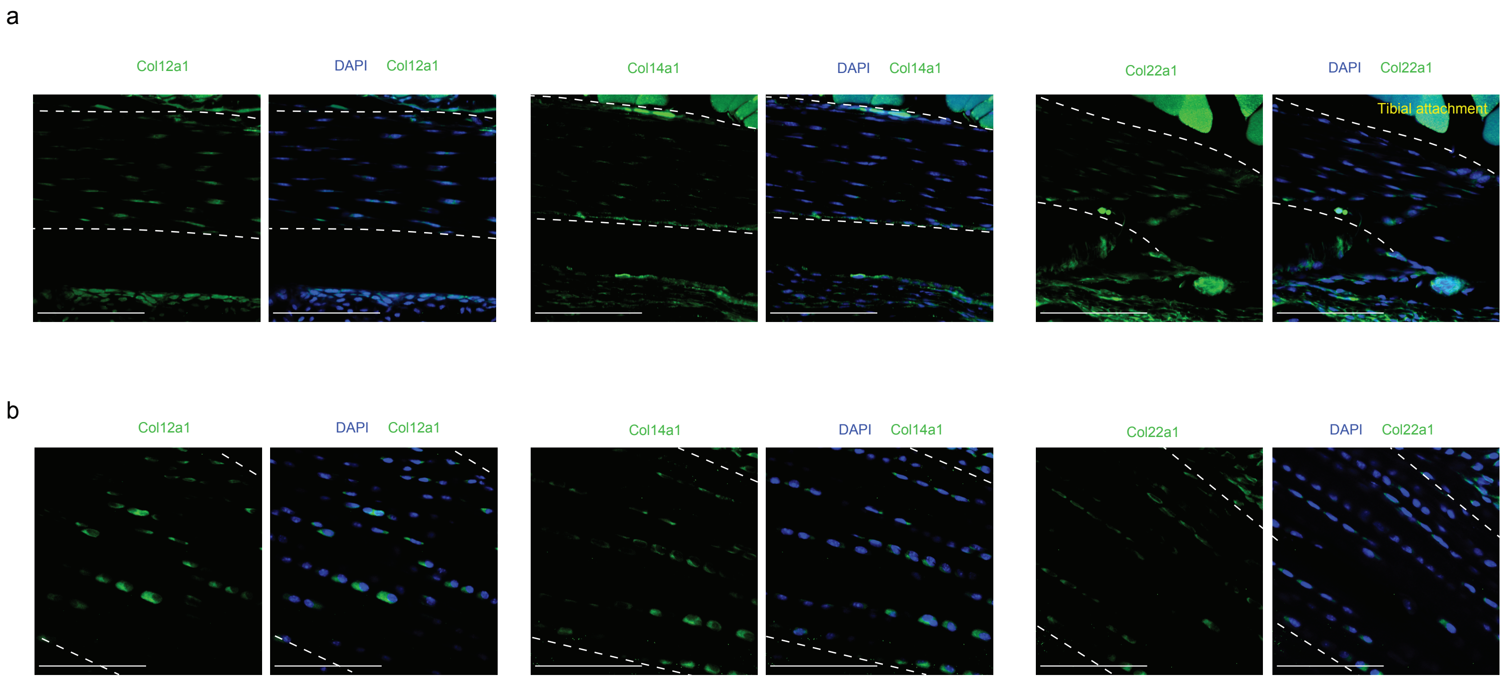

Supplementary Fig. 2 RNA in situ hybridization images.

a, b RNA in situ hybridization of Col12a1, Col14a1 and Col22a1 in the MCL (a) and ACL (b).

Scale bar: 100 μm.

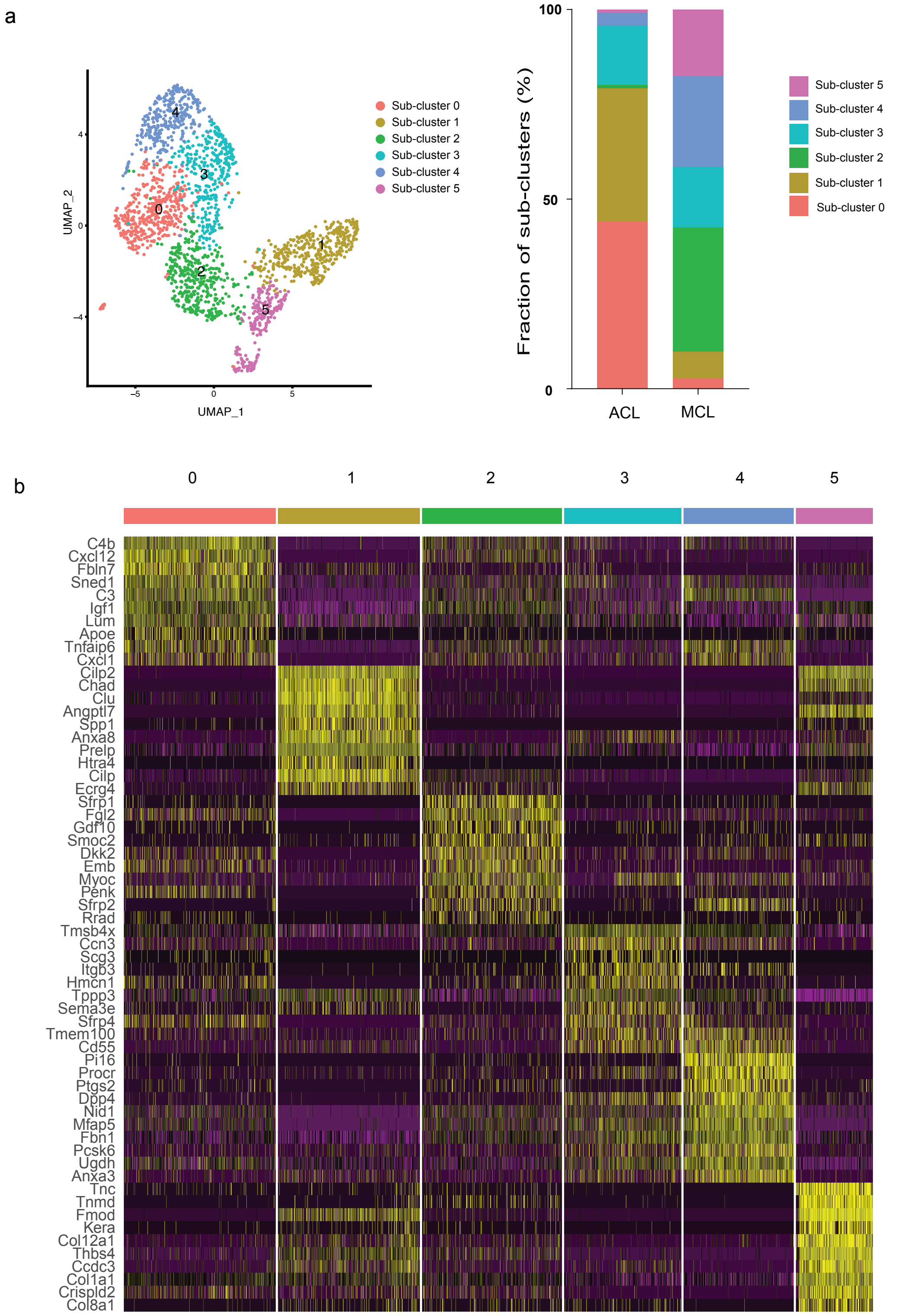

Supplementary Fig. 3 Further investigation of subpopulations of *Col12a1*- and *Col14a1*- positive cells.

a Left, UMAP plots for sub-clusters of *Col14a1*- and *Col12a1*-positive cells in the ACL and MCL. Right, fraction of each sub-cluster in the ACL and MCL. b Heatmap showing the marker genes for each sub-cluster.

Ishibashi K, et al

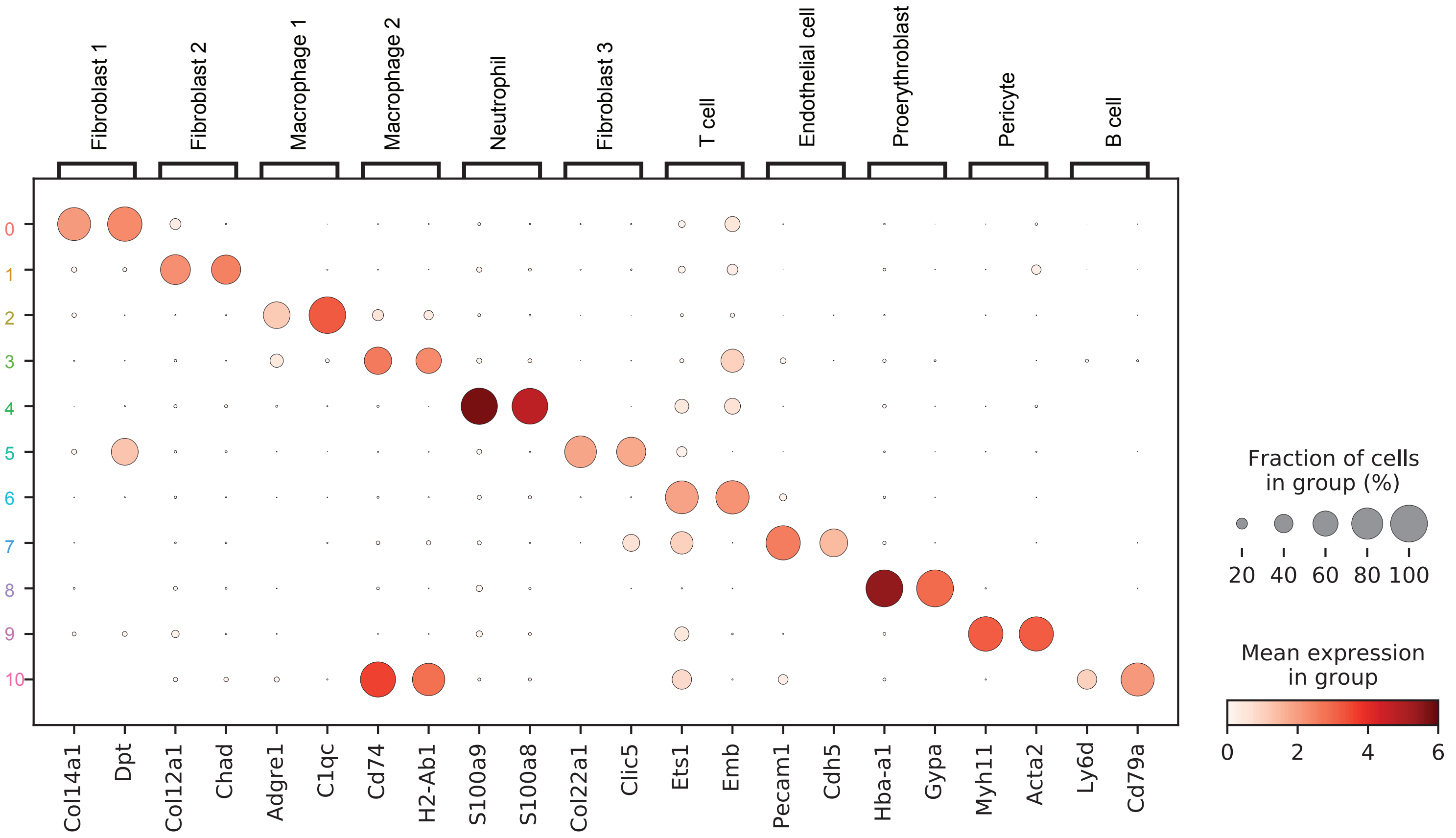

Supplementary Fig. 4 Cell annotation for ligament cells of young and aged mice.

Marker genes for each cluster are shown. Dot size represents the percentage of cells expressing a gene within a cluster. The intensity of the dot color indicates the mean expression level.
