# Supplementary material for: Single-cell transcriptome analysis reveals cellular heterogeneity in mouse intra- and extra articular ligaments
Source: Commun Biol. 2022 Nov 12;5:1233. doi: 10.1038/s42003-022-04196-w (PMC9653455; doi:10.1038/s42003-022-04196-w)
Supplement: Supplementary file 3 — Description of Additional Supplementary Data [file 42003_2022_4196_MOESM3_ESM.docx]

**Description of Additional Supplementary Files**

**File name:** Supplementary Data 1

**Description:** Source data for graphs in the paper

**File name:** Supplementary Data 2

**Description:** Source data for gene ontology analysis showing upregulated pathways
